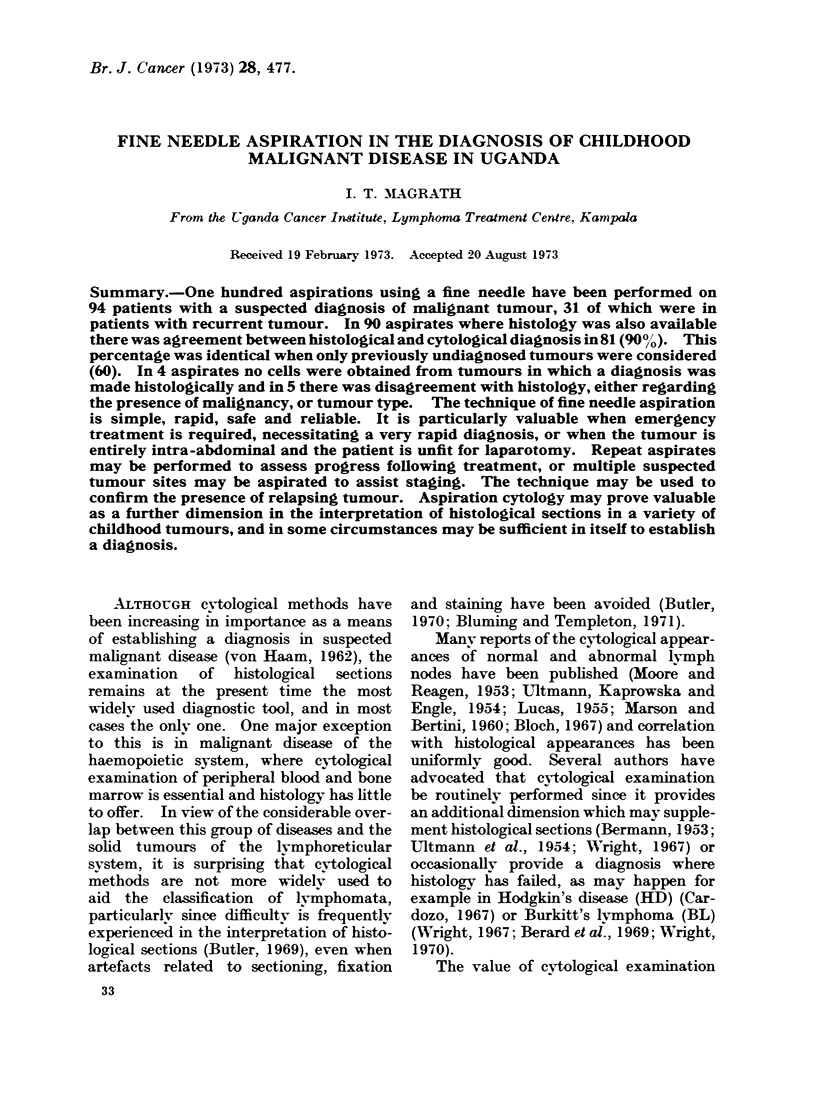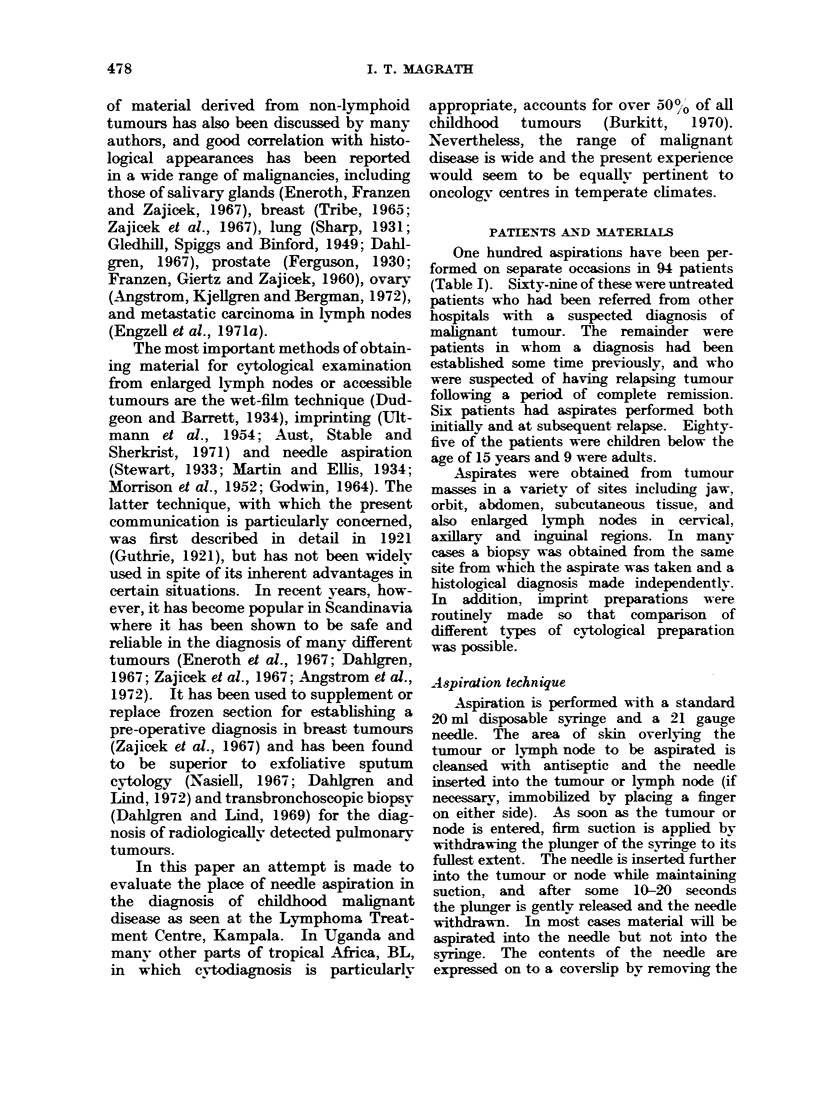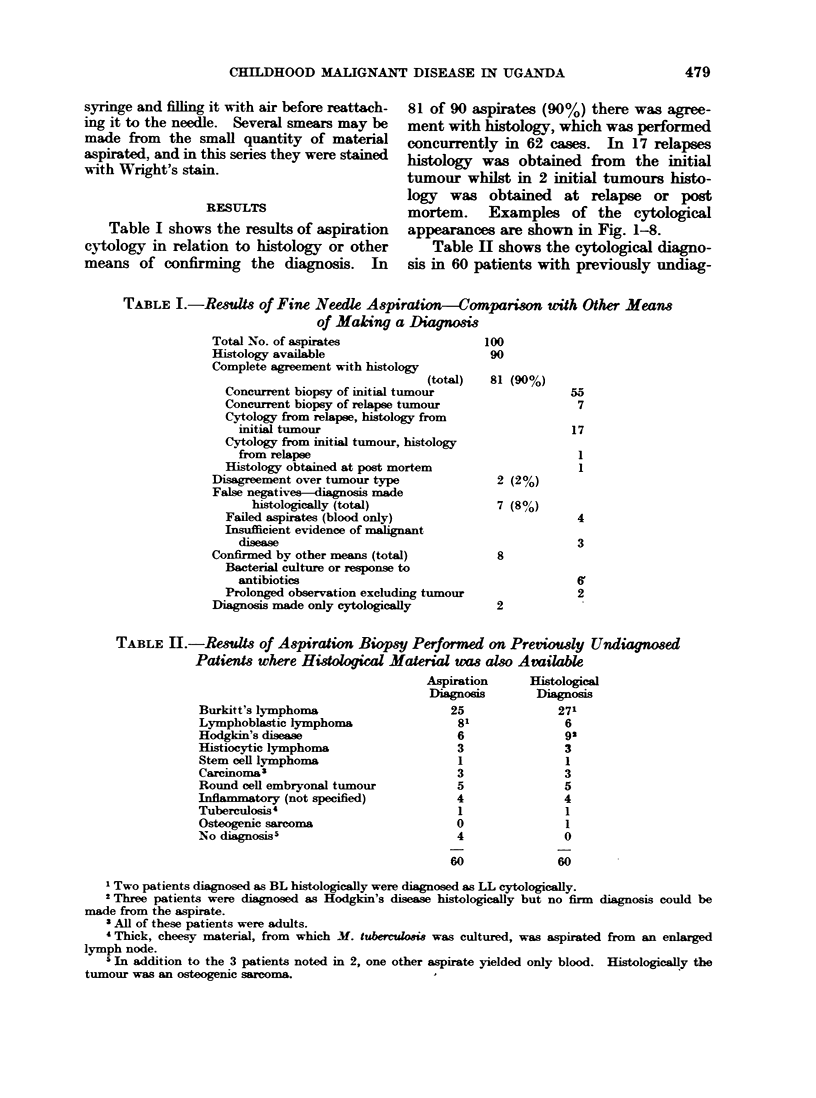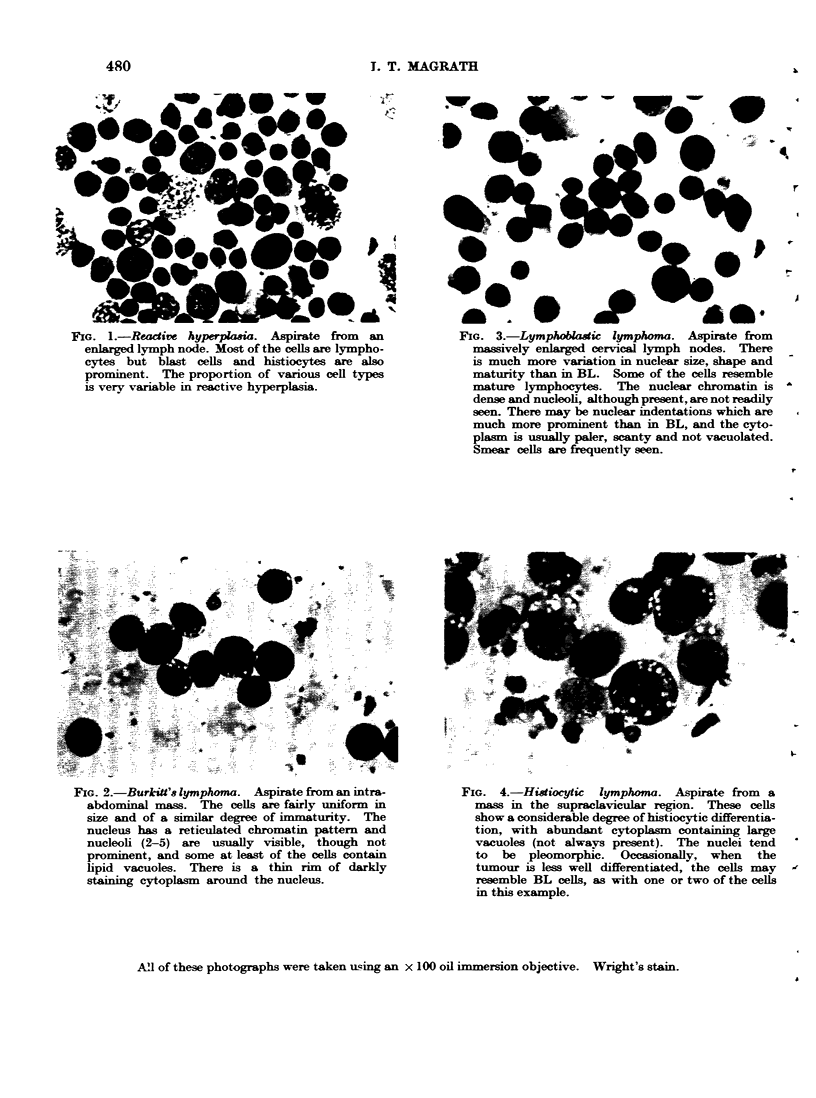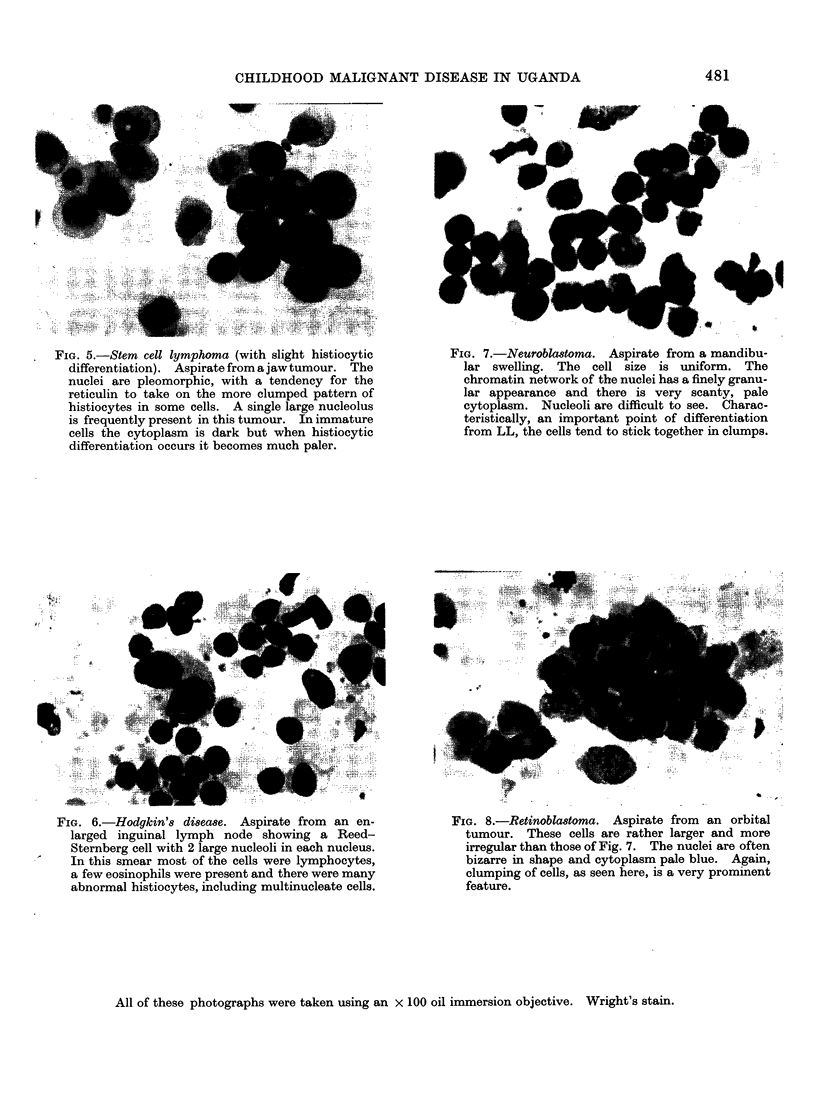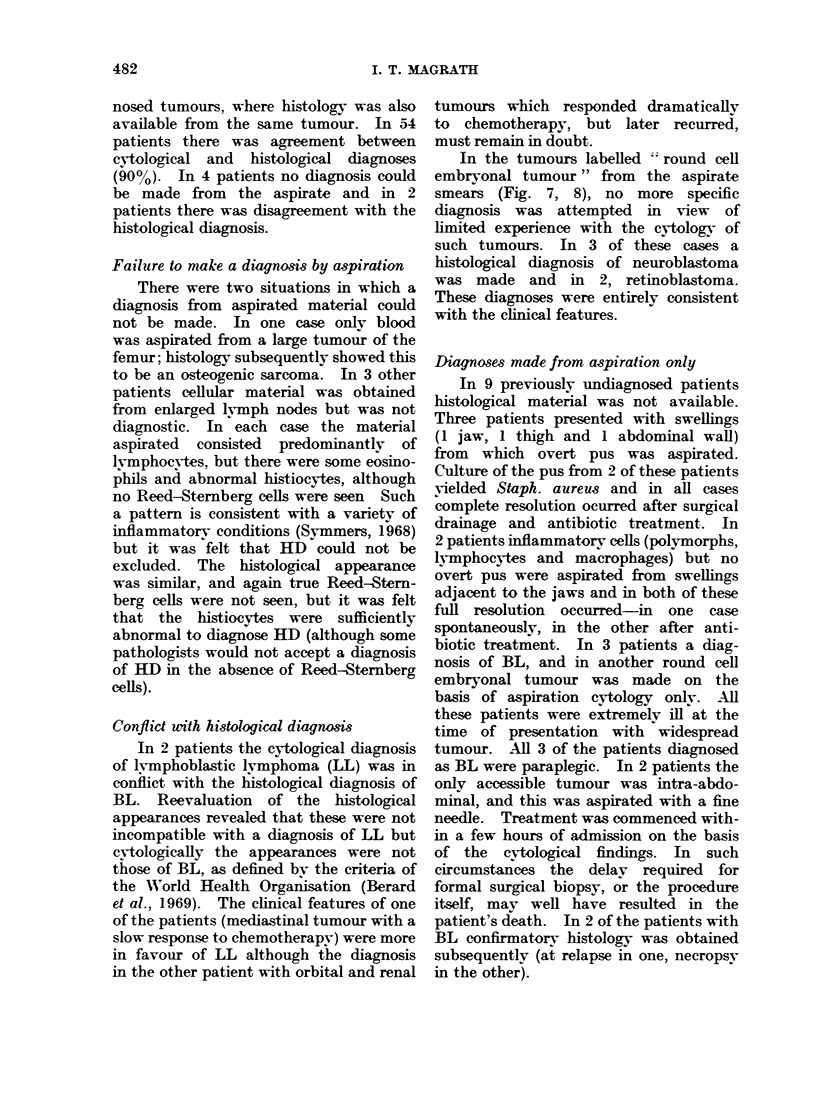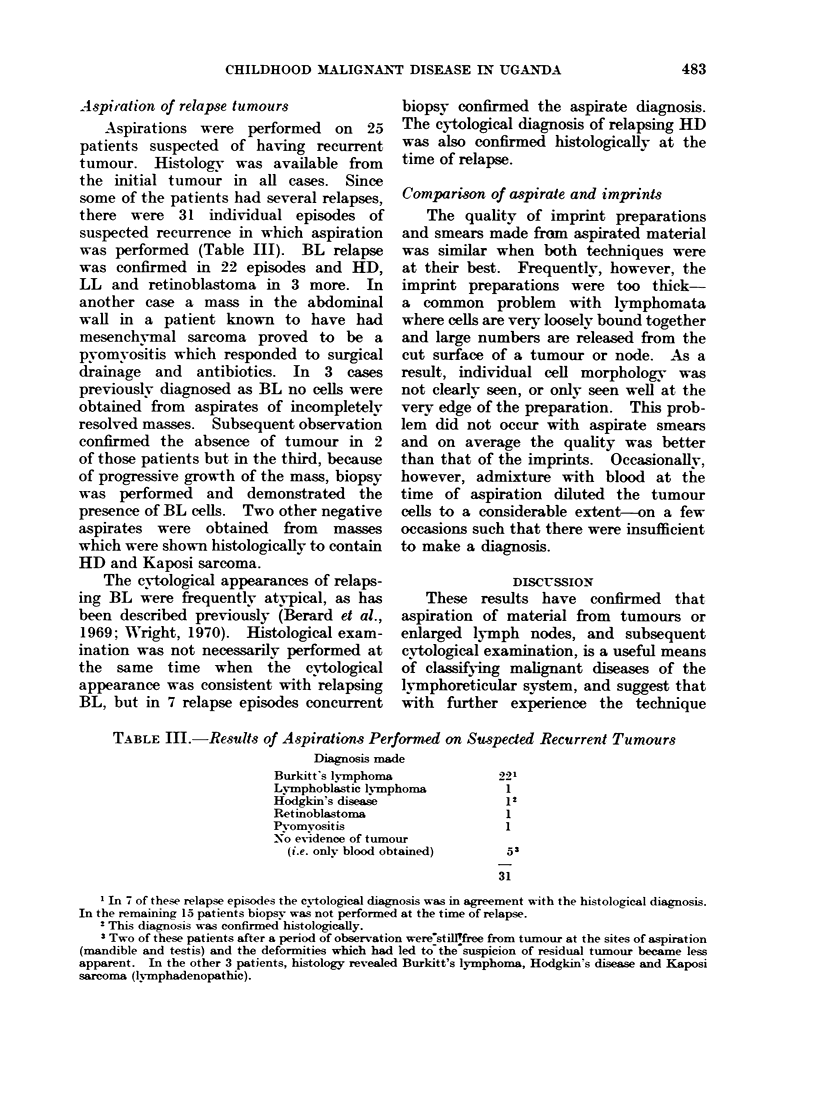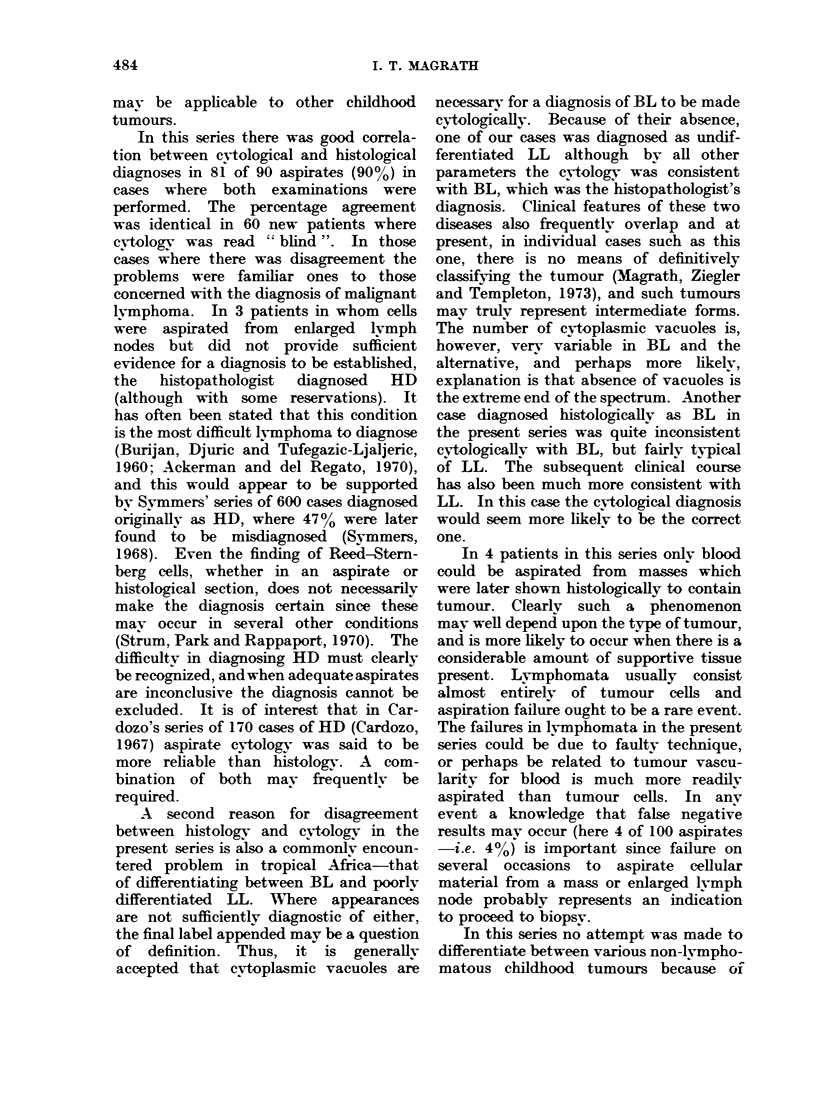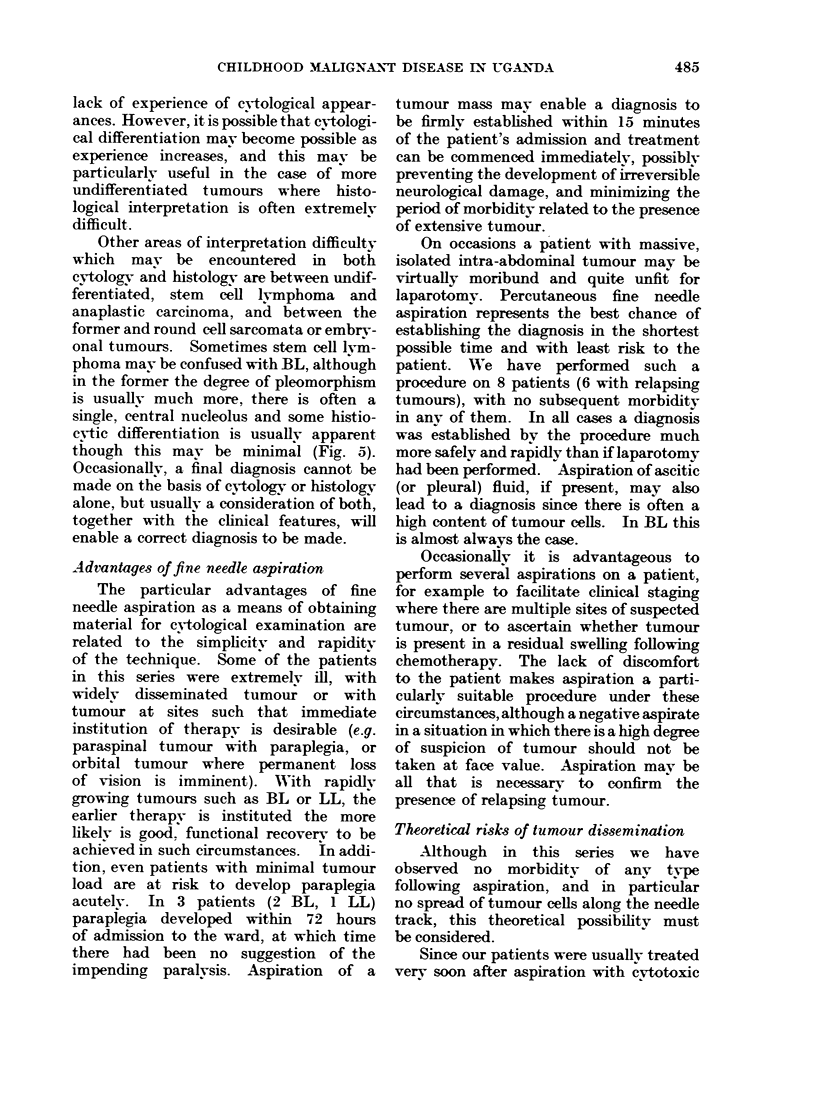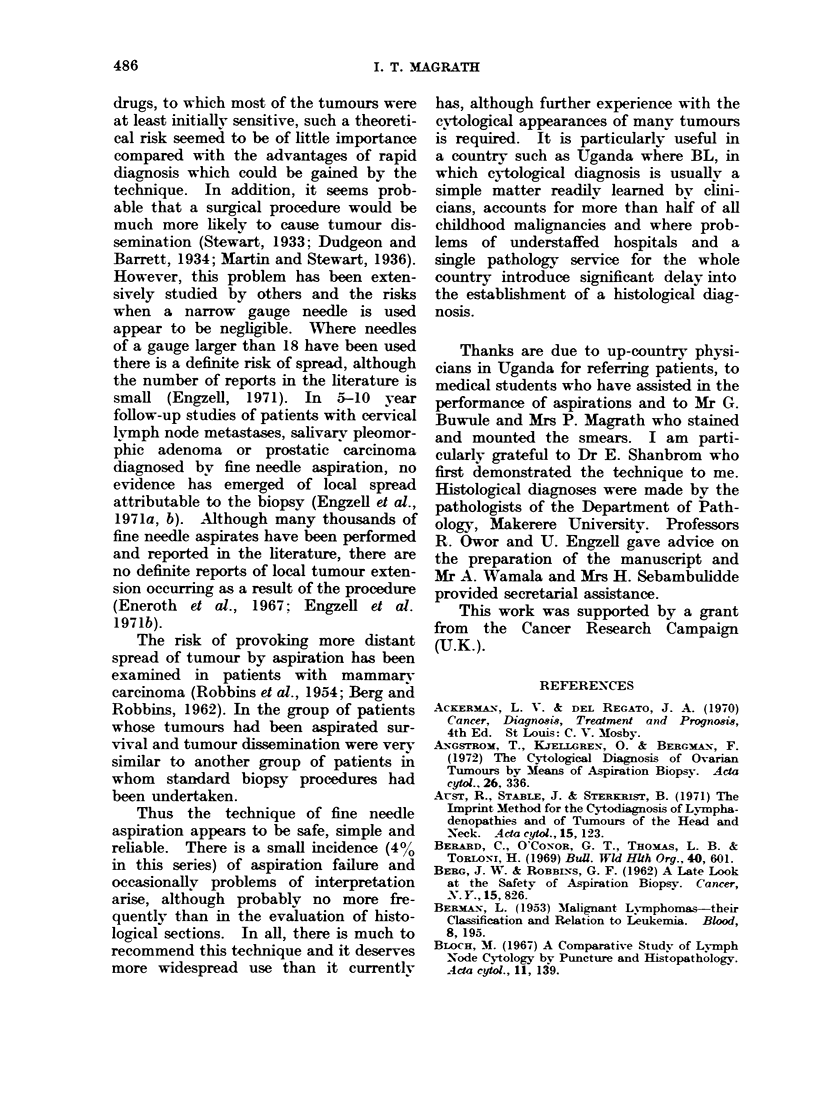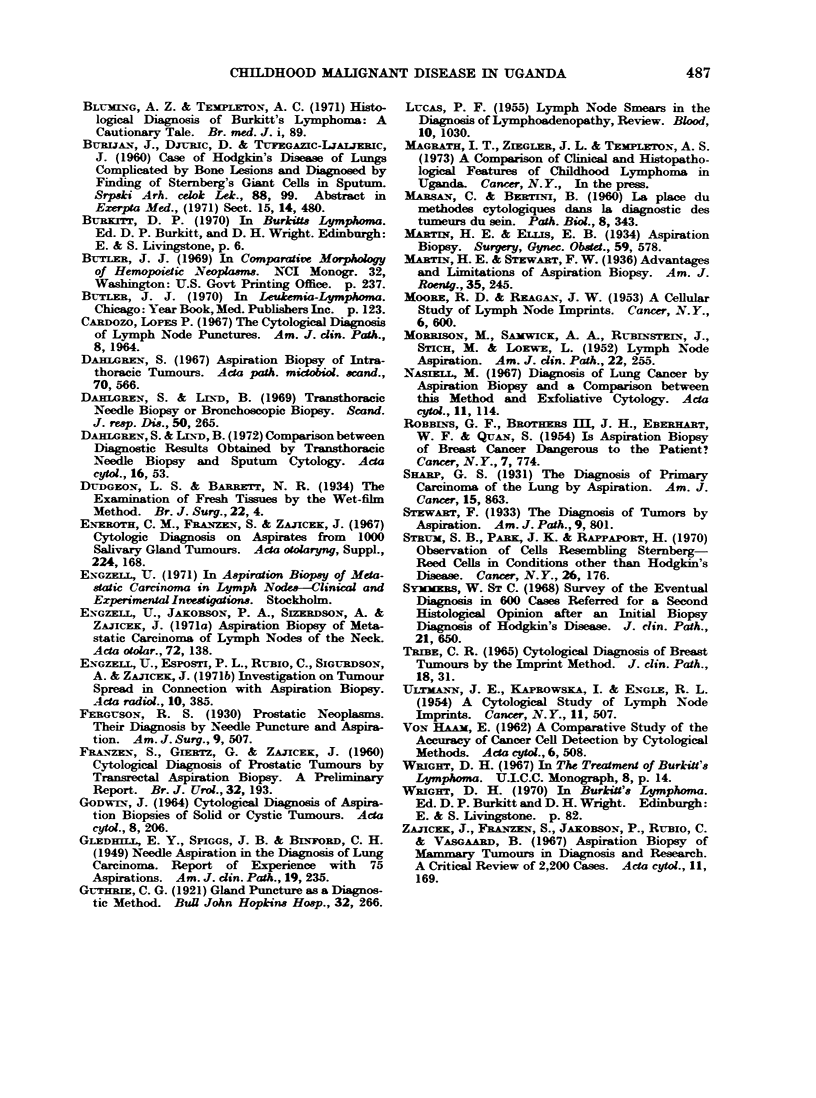# Fine Needle Aspiration in the Diagnosis of Childhood Malignant Disease in Uganda

**DOI:** 10.1038/bjc.1973.177

**Published:** 1973-12

**Authors:** I. T. Magrath

## Abstract

**Images:**


*Br. J. Cancer* (1973) **28**, 477.

### FINE NEEDLE ASPIRATION IN THE DIAGNOSIS OF CHILDHOOD MALIGNANT DISEASE IN UGANDA

I. T. MAGRATH

*From the Uganda Cancer Institute, Lymphoma Treatment Centre, Kampala*

Received 19 February 1973. Accepted 20 August 1973

**Summary.—One hundred aspirations using a fine needle have been performed on 94 patients with a suspected diagnosis of malignant tumour, 31 of which were in patients with recurrent tumour. In 90 aspirates where histology was also available there was agreement between histological and cytological diagnosis in 81 (90%). This percentage was identical when only previously undiagnosed tumours were considered (60). In 4 aspirates no cells were obtained from tumours in which a diagnosis was made histologically and in 5 there was disagreement with histology, either regarding the presence of malignancy, or tumour type. The technique of fine needle aspiration is simple, rapid, safe and reliable. It is particularly valuable when emergency treatment is required, necessitating a very rapid diagnosis, or when the tumour is entirely intra-abdominal and the patient is unfit for laparotomy. Repeat aspirates may be performed to assess progress following treatment, or multiple suspected tumour sites may be aspirated to assist staging. The technique may be used to confirm the presence of relapsing tumour. Aspiration cytology may prove valuable as a further dimension in the interpretation of histological sections in a variety of childhood tumours, and in some circumstances may be sufficient in itself to establish a diagnosis.**

Although cytological methods have been increasing in importance as a means of establishing a diagnosis in suspected malignant disease (von Haam, 1962), the examination of histological sections remains at the present time the most widely used diagnostic tool, and in most cases the only one. One major exception to this is in malignant disease of the haemopoietic system, where cytological examination of peripheral blood and bone marrow is essential and histology has little to offer. In view of the considerable overlap between this group of diseases and the solid tumours of the lymphoreticular system, it is surprising that cytological methods are not more widely used to aid the classification of lymphomata, particularly since difficulty is frequently experienced in the interpretation of histological sections (Butler, 1969), even when artefacts related to sectioning, fixation and staining have been avoided (Butler, 1970; Bluming and Templeton, 1971).

Many reports of the cytological appearances of normal and abnormal lymph nodes have been published (Moore and Reagen, 1953; Ultmann, Kaprowska and Engle, 1954; Lucas, 1955; Marson and Bertini, 1960; Bloch, 1967) and correlation with histological appearances has been uniformly good. Several authors have advocated that cytological examination be routinely performed since it provides an additional dimension which may supplement histological sections (Bermann, 1953; Ultmann *et al.*, 1954; Wright, 1967) or occasionally provide a diagnosis where histology has failed, as may happen for example in Hodgkin's disease (HD) (Cardozo, 1967) or Burkitt's lymphoma (BL) (Wright, 1967; Berard *et al.*, 1969; Wright, 1970).

The value of cytological examination of material derived from non-lymphoid tumours has also been discussed by many authors, and good correlation with histological appearances has been reported in a wide range of malignancies, including those of salivary glands (Eneroth, Franzen and Zajicek, 1967), breast (Tribe, 1965; Zajicek *et al.*, 1967), lung (Sharp, 1931; Gledhill, Spiggs and Binford, 1949; Dahlgren, 1967), prostate (Ferguson, 1930; Franzen, Giertz and Zajicek, 1960), ovary (Angstrom, Kjellgren and Bergman, 1972), and metastatic carcinoma in lymph nodes (Engzell *et al.*, 1971*a*).

The most important methods of obtaining material for cytological examination from enlarged lymph nodes or accessible tumours are the wet-film technique (Dudgeon and Barrett, 1934), imprinting (Ultmann *et al.*, 1954; Aust, Stable and Sherkrist, 1971) and needle aspiration (Stewart, 1933; Martin and Ellis, 1934; Morrison *et al.*, 1952; Godwin, 1964). The latter technique, with which the present communication is particularly concerned, was first described in detail in 1921 (Guthrie, 1921), but has not been widely used in spite of its inherent advantages in certain situations. In recent years, however, it has become popular in Scandinavia where it has been shown to be safe and reliable in the diagnosis of many different tumours (Eneroth *et al.*, 1967; Dahlgren, 1967; Zajicek *et al.*, 1967; Angstrom *et al.*, 1972). It has been used to supplement or replace frozen section for establishing a pre-operative diagnosis in breast tumours (Zajicek *et al.*, 1967) and has been found to be superior to exfoliative sputum cytology (Nasiell, 1967; Dahlgren and Lind, 1972) and transbronchoscopic biopsy (Dahlgren and Lind, 1969) for the diagnosis of radiologically detected pulmonary tumours.

In this paper an attempt is made to evaluate the place of needle aspiration in the diagnosis of childhood malignant disease as seen at the Lymphoma Treatment Centre, Kampala. In Uganda and many other parts of tropical Africa, BL, in which cytodiagnosis is particularly appropriate, accounts for over 50% of all childhood tumours (Burkitt, 1970). Nevertheless, the range of malignant disease is wide and the present experience would seem to be equally pertinent to oncology centres in temperate climates.

### PATIENTS AND MATERIALS

One hundred aspirations have been performed on separate occasions in 94 patients (Table I). Sixty-nine of these were untreated patients who had been referred from other hospitals with a suspected diagnosis of malignant tumour. The remainder were patients in whom a diagnosis had been established some time previously, and who were suspected of having relapsing tumour following a period of complete remission. Six patients had aspirates performed both initially and at subsequent relapse. Eighty-five of the patients were children below the age of 15 years and 9 were adults.

Aspirates were obtained from tumour masses in a variety of sites including jaw, orbit, abdomen, subcutaneous tissue, and also enlarged lymph nodes in cervical, axillary and inguinal regions. In many cases a biopsy was obtained from the same site from which the aspirate was taken and a histological diagnosis made independently. In addition, imprint preparations were routinely made so that comparison of different types of cytological preparation was possible.

### *Aspiration technique*

Aspiration is performed with a standard 20 ml disposable syringe and a 21 gauge needle. The area of skin overlying the tumour or lymph node to be aspirated is cleansed with antiseptic and the needle inserted into the tumour or lymph node (if necessary, immobilized by placing a finger on either side). As soon as the tumour or node is entered, firm suction is applied by withdrawing the plunger of the syringe to its fullest extent. The needle is inserted further into the tumour or node while maintaining suction, and after some 10–20 seconds the plunger is gently released and the needle withdrawn. In most cases material will be aspirated into the needle but not into the syringe. The contents of the needle are expressed on to a coverslip by removing the syringe and filling it with air before reattaching it to the needle. Several smears may be made from the small quantity of material aspirated, and in this series they were stained with Wright's stain.

### RESULTS

Table I shows the results of aspiration cytology in relation to histology or other means of confirming the diagnosis. In 81 of 90 aspirates (90%) there was agreement with histology, which was performed concurrently in 62 cases. In 17 relapses histology was obtained from the initial tumour whilst in 2 initial tumours histology was obtained at relapse or post mortem. Examples of the cytological appearances are shown in Fig. 1–8.

Table II shows the cytological diagnosis in 60 patients with previously undiag-

TABLE I.—*Results of Fine Needle Aspiration—Comparison with Other Means of Making a Diagnosis*

| | | |
|---|---|---|
| Total No. of aspirates | 100 | |
| Histology available | 90 | |
| Complete agreement with histology (total) | 81 (90%) | |
| Concurrent biopsy of initial tumour | | 55 |
| Concurrent biopsy of relapse tumour | | 7 |
| Cytology from relapse, histology from initial tumour | | 17 |
| Cytology from initial tumour, histology from relapse | | 1 |
| Histology obtained at post mortem | | 1 |
| Disagreement over tumour type | 2 (2%) | |
| False negatives—diagnosis made histologically (total) | 7 (8%) | |
| Failed aspirates (blood only) | | 4 |
| Insufficient evidence of malignant disease | | 3 |
| Confirmed by other means (total) | 8 | |
| Bacterial culture or response to antibiotics | | 6 |
| Prolonged observation excluding tumour | | 2 |
| Diagnosis made only cytologically | 2 | |

TABLE II.—*Results of Aspiration Biopsy Performed on Previously Undiagnosed Patients where Histological Material was also Available*

| | Aspiration Diagnosis | Histological Diagnosis |
|---|---|---|
| Burkitt's lymphoma | 25 | 27[1] |
| Lymphoblastic lymphoma | 8[1] | 6 |
| Hodgkin's disease | 6 | 9[2] |
| Histiocytic lymphoma | 3 | 3 |
| Stem cell lymphoma | 1 | 1 |
| Carcinoma[3] | 3 | 3 |
| Round cell embryonal tumour | 5 | 5 |
| Inflammatory (not specified) | 4 | 4 |
| Tuberculosis[4] | 1 | 1 |
| Osteogenic sarcoma | 0 | 1 |
| No diagnosis[5] | 4 | 0 |
| | 60 | 60 |

[1] Two patients diagnosed as BL histologically were diagnosed as LL cytologically.
[2] Three patients were diagnosed as Hodgkin's disease histologically but no firm diagnosis could be made from the aspirate.
[3] All of these patients were adults.
[4] Thick, cheesy material, from which *M. tuberculosis* was cultured, was aspirated from an enlarged lymph node.
[5] In addition to the 3 patients noted in 2, one other aspirate yielded only blood. Histologically the tumour was an osteogenic sarcoma.

FIG. 1.—*Reactive hyperplasia.* Aspirate from an enlarged lymph node. Most of the cells are lymphocytes but blast cells and histiocytes are also prominent. The proportion of various cell types is very variable in reactive hyperplasia.

FIG. 3.—*Lymphoblastic lymphoma.* Aspirate from massively enlarged cervical lymph nodes. There is much more variation in nuclear size, shape and maturity than in BL. Some of the cells resemble mature lymphocytes. The nuclear chromatin is dense and nucleoli, although present, are not readily seen. There may be nuclear indentations which are much more prominent than in BL, and the cytoplasm is usually paler, scanty and not vacuolated. Smear cells are frequently seen.

FIG. 2.—*Burkitt's lymphoma.* Aspirate from an intra-abdominal mass. The cells are fairly uniform in size and of a similar degree of immaturity. The nucleus has a reticulated chromatin pattern and nucleoli (2–5) are usually visible, though not prominent, and some at least of the cells contain lipid vacuoles. There is a thin rim of darkly staining cytoplasm around the nucleus.

FIG. 4.—*Histiocytic lymphoma.* Aspirate from a mass in the supraclavicular region. These cells show a considerable degree of histiocytic differentiation, with abundant cytoplasm containing large vacuoles (not always present). The nuclei tend to be pleomorphic. Occasionally, when the tumour is less well differentiated, the cells may resemble BL cells, as with one or two of the cells in this example.

All of these photographs were taken using an $\times 100$ oil immersion objective. Wright's stain.

FIG. 5.—*Stem cell lymphoma* (with slight histiocytic differentiation). Aspirate from a jaw tumour. The nuclei are pleomorphic, with a tendency for the reticulin to take on the more clumped pattern of histiocytes in some cells. A single large nucleolus is frequently present in this tumour. In immature cells the cytoplasm is dark but when histiocytic differentiation occurs it becomes much paler.

FIG. 7.—*Neuroblastoma.* Aspirate from a mandibular swelling. The cell size is uniform. The chromatin network of the nuclei has a finely granular appearance and there is very scanty, pale cytoplasm. Nucleoli are difficult to see. Characteristically, an important point of differentiation from LL, the cells tend to stick together in clumps.

FIG. 6.—*Hodgkin's disease.* Aspirate from an enlarged inguinal lymph node showing a Reed–Sternberg cell with 2 large nucleoli in each nucleus. In this smear most of the cells were lymphocytes, a few eosinophils were present and there were many abnormal histiocytes, including multinucleate cells.

FIG. 8.—*Retinoblastoma.* Aspirate from an orbital tumour. These cells are rather larger and more irregular than those of Fig. 7. The nuclei are often bizarre in shape and cytoplasm pale blue. Again, clumping of cells, as seen here, is a very prominent feature.

All of these photographs were taken using an $\times 100$ oil immersion objective. Wright's stain.

nosed tumours, where histology was also available from the same tumour. In 54 patients there was agreement between cytological and histological diagnoses (90%). In 4 patients no diagnosis could be made from the aspirate and in 2 patients there was disagreement with the histological diagnosis.

*Failure to make a diagnosis by aspiration*

There were two situations in which a diagnosis from aspirated material could not be made. In one case only blood was aspirated from a large tumour of the femur; histology subsequently showed this to be an osteogenic sarcoma. In 3 other patients cellular material was obtained from enlarged lymph nodes but was not diagnostic. In each case the material aspirated consisted predominantly of lymphocytes, but there were some eosinophils and abnormal histiocytes, although no Reed–Sternberg cells were seen Such a pattern is consistent with a variety of inflammatory conditions (Symmers, 1968) but it was felt that HD could not be excluded. The histological appearance was similar, and again true Reed–Sternberg cells were not seen, but it was felt that the histiocytes were sufficiently abnormal to diagnose HD (although some pathologists would not accept a diagnosis of HD in the absence of Reed–Sternberg cells).

*Conflict with histological diagnosis*

In 2 patients the cytological diagnosis of lymphoblastic lymphoma (LL) was in conflict with the histological diagnosis of BL. Reevaluation of the histological appearances revealed that these were not incompatible with a diagnosis of LL but cytologically the appearances were not those of BL, as defined by the criteria of the World Health Organisation (Berard *et al.*, 1969). The clinical features of one of the patients (mediastinal tumour with a slow response to chemotherapy) were more in favour of LL although the diagnosis in the other patient with orbital and renal tumours which responded dramatically to chemotherapy, but later recurred, must remain in doubt.

In the tumours labelled "round cell embryonal tumour" from the aspirate smears (Fig. 7, 8), no more specific diagnosis was attempted in view of limited experience with the cytology of such tumours. In 3 of these cases a histological diagnosis of neuroblastoma was made and in 2, retinoblastoma. These diagnoses were entirely consistent with the clinical features.

*Diagnoses made from aspiration only*

In 9 previously undiagnosed patients histological material was not available. Three patients presented with swellings (1 jaw, 1 thigh and 1 abdominal wall) from which overt pus was aspirated. Culture of the pus from 2 of these patients yielded *Staph. aureus* and in all cases complete resolution ocurred after surgical drainage and antibiotic treatment. In 2 patients inflammatory cells (polymorphs, lymphocytes and macrophages) but no overt pus were aspirated from swellings adjacent to the jaws and in both of these full resolution occurred—in one case spontaneously, in the other after antibiotic treatment. In 3 patients a diagnosis of BL, and in another round cell embryonal tumour was made on the basis of aspiration cytology only. All these patients were extremely ill at the time of presentation with widespread tumour. All 3 of the patients diagnosed as BL were paraplegic. In 2 patients the only accessible tumour was intra-abdominal, and this was aspirated with a fine needle. Treatment was commenced within a few hours of admission on the basis of the cytological findings. In such circumstances the delay required for formal surgical biopsy, or the procedure itself, may well have resulted in the patient's death. In 2 of the patients with BL confirmatory histology was obtained subsequently (at relapse in one, necropsy in the other).

*Aspiration of relapse tumours*

Aspirations were performed on 25 patients suspected of having recurrent tumour. Histology was available from the initial tumour in all cases. Since some of the patients had several relapses, there were 31 individual episodes of suspected recurrence in which aspiration was performed (Table III). BL relapse was confirmed in 22 episodes and HD, LL and retinoblastoma in 3 more. In another case a mass in the abdominal wall in a patient known to have had mesenchymal sarcoma proved to be a pyomyositis which responded to surgical drainage and antibiotics. In 3 cases previously diagnosed as BL no cells were obtained from aspirates of incompletely resolved masses. Subsequent observation confirmed the absence of tumour in 2 of those patients but in the third, because of progressive growth of the mass, biopsy was performed and demonstrated the presence of BL cells. Two other negative aspirates were obtained from masses which were shown histologically to contain HD and Kaposi sarcoma.

The cytological appearances of relapsing BL were frequently atypical, as has been described previously (Berard *et al.*, 1969; Wright, 1970). Histological examination was not necessarily performed at the same time when the cytological appearance was consistent with relapsing BL, but in 7 relapse episodes concurrent biopsy confirmed the aspirate diagnosis. The cytological diagnosis of relapsing HD was also confirmed histologically at the time of relapse.

*Comparison of aspirate and imprints*

The quality of imprint preparations and smears made from aspirated material was similar when both techniques were at their best. Frequently, however, the imprint preparations were too thick—a common problem with lymphomata where cells are very loosely bound together and large numbers are released from the cut surface of a tumour or node. As a result, individual cell morphology was not clearly seen, or only seen well at the very edge of the preparation. This problem did not occur with aspirate smears and on average the quality was better than that of the imprints. Occasionally, however, admixture with blood at the time of aspiration diluted the tumour cells to a considerable extent—on a few occasions such that there were insufficient to make a diagnosis.

### DISCUSSION

These results have confirmed that aspiration of material from tumours or enlarged lymph nodes, and subsequent cytological examination, is a useful means of classifying malignant diseases of the lymphoreticular system, and suggest that with further experience the technique

TABLE III.—*Results of Aspirations Performed on Suspected Recurrent Tumours*

| Diagnosis made | |
|---|---|
| Burkitt's lymphoma | 22[1] |
| Lymphoblastic lymphoma | 1 |
| Hodgkin's disease | 1[2] |
| Retinoblastoma | 1 |
| Pyomyositis | 1 |
| No evidence of tumour (*i.e.* only blood obtained) | 5[3] |
| | 31 |

[1] In 7 of these relapse episodes the cytological diagnosis was in agreement with the histological diagnosis. In the remaining 15 patients biopsy was not performed at the time of relapse.

[2] This diagnosis was confirmed histologically.

[3] Two of these patients after a period of observation were still free from tumour at the sites of aspiration (mandible and testis) and the deformities which had led to the suspicion of residual tumour became less apparent. In the other 3 patients, histology revealed Burkitt's lymphoma, Hodgkin's disease and Kaposi sarcoma (lymphadenopathic).

may be applicable to other childhood tumours.

In this series there was good correlation between cytological and histological diagnoses in 81 of 90 aspirates (90%) in cases where both examinations were performed. The percentage agreement was identical in 60 new patients where cytology was read "blind". In those cases where there was disagreement the problems were familiar ones to those concerned with the diagnosis of malignant lymphoma. In 3 patients in whom cells were aspirated from enlarged lymph nodes but did not provide sufficient evidence for a diagnosis to be established, the histopathologist diagnosed HD (although with some reservations). It has often been stated that this condition is the most difficult lymphoma to diagnose (Burijan, Djuric and Tufegazic-Ljaljeric, 1960; Ackerman and del Regato, 1970), and this would appear to be supported by Symmers' series of 600 cases diagnosed originally as HD, where 47% were later found to be misdiagnosed (Symmers, 1968). Even the finding of Reed–Sternberg cells, whether in an aspirate or histological section, does not necessarily make the diagnosis certain since these may occur in several other conditions (Strum, Park and Rappaport, 1970). The difficulty in diagnosing HD must clearly be recognized, and when adequate aspirates are inconclusive the diagnosis cannot be excluded. It is of interest that in Cardozo's series of 170 cases of HD (Cardozo, 1967) aspirate cytology was said to be more reliable than histology. A combination of both may frequently be required.

A second reason for disagreement between histology and cytology in the present series is also a commonly encountered problem in tropical Africa—that of differentiating between BL and poorly differentiated LL. Where appearances are not sufficiently diagnostic of either, the final label appended may be a question of definition. Thus, it is generally accepted that cytoplasmic vacuoles are necessary for a diagnosis of BL to be made cytologically. Because of their absence, one of our cases was diagnosed as undifferentiated LL although by all other parameters the cytology was consistent with BL, which was the histopathologist's diagnosis. Clinical features of these two diseases also frequently overlap and at present, in individual cases such as this one, there is no means of definitively classifying the tumour (Magrath, Ziegler and Templeton, 1973), and such tumours may truly represent intermediate forms. The number of cytoplasmic vacuoles is, however, very variable in BL and the alternative, and perhaps more likely, explanation is that absence of vacuoles is the extreme end of the spectrum. Another case diagnosed histologically as BL in the present series was quite inconsistent cytologically with BL, but fairly typical of LL. The subsequent clinical course has also been much more consistent with LL. In this case the cytological diagnosis would seem more likely to be the correct one.

In 4 patients in this series only blood could be aspirated from masses which were later shown histologically to contain tumour. Clearly such a phenomenon may well depend upon the type of tumour, and is more likely to occur when there is a considerable amount of supportive tissue present. Lymphomata usually consist almost entirely of tumour cells and aspiration failure ought to be a rare event. The failures in lymphomata in the present series could be due to faulty technique, or perhaps be related to tumour vascularity for blood is much more readily aspirated than tumour cells. In any event a knowledge that false negative results may occur (here 4 of 100 aspirates —*i.e.* 4%) is important since failure on several occasions to aspirate cellular material from a mass or enlarged lymph node probably represents an indication to proceed to biopsy.

In this series no attempt was made to differentiate between various non-lymphomatous childhood tumours because of lack of experience of cytological appearances. However, it is possible that cytological differentiation may become possible as experience increases, and this may be particularly useful in the case of more undifferentiated tumours where histological interpretation is often extremely difficult.

Other areas of interpretation difficulty which may be encountered in both cytology and histology are between undifferentiated, stem cell lymphoma and anaplastic carcinoma, and between the former and round cell sarcomata or embryonal tumours. Sometimes stem cell lymphoma may be confused with BL, although in the former the degree of pleomorphism is usually much more, there is often a single, central nucleolus and some histiocytic differentiation is usually apparent though this may be minimal (Fig. 5). Occasionally, a final diagnosis cannot be made on the basis of cytology or histology alone, but usually a consideration of both, together with the clinical features, will enable a correct diagnosis to be made.

*Advantages of fine needle aspiration*

The particular advantages of fine needle aspiration as a means of obtaining material for cytological examination are related to the simplicity and rapidity of the technique. Some of the patients in this series were extremely ill, with widely disseminated tumour or with tumour at sites such that immediate institution of therapy is desirable (*e.g.* paraspinal tumour with paraplegia, or orbital tumour where permanent loss of vision is imminent). With rapidly growing tumours such as BL or LL, the earlier therapy is instituted the more likely is good, functional recovery to be achieved in such circumstances. In addition, even patients with minimal tumour load are at risk to develop paraplegia acutely. In 3 patients (2 BL, 1 LL) paraplegia developed within 72 hours of admission to the ward, at which time there had been no suggestion of the impending paralysis. Aspiration of a tumour mass may enable a diagnosis to be firmly established within 15 minutes of the patient's admission and treatment can be commenced immediately, possibly preventing the development of irreversible neurological damage, and minimizing the period of morbidity related to the presence of extensive tumour.

On occasions a patient with massive, isolated intra-abdominal tumour may be virtually moribund and quite unfit for laparotomy. Percutaneous fine needle aspiration represents the best chance of establishing the diagnosis in the shortest possible time and with least risk to the patient. We have performed such a procedure on 8 patients (6 with relapsing tumours), with no subsequent morbidity in any of them. In all cases a diagnosis was established by the procedure much more safely and rapidly than if laparotomy had been performed. Aspiration of ascitic (or pleural) fluid, if present, may also lead to a diagnosis since there is often a high content of tumour cells. In BL this is almost always the case.

Occasionally it is advantageous to perform several aspirations on a patient, for example to facilitate clinical staging where there are multiple sites of suspected tumour, or to ascertain whether tumour is present in a residual swelling following chemotherapy. The lack of discomfort to the patient makes aspiration a particularly suitable procedure under these circumstances, although a negative aspirate in a situation in which there is a high degree of suspicion of tumour should not be taken at face value. Aspiration may be all that is necessary to confirm the presence of relapsing tumour.

*Theoretical risks of tumour dissemination*

Although in this series we have observed no morbidity of any type following aspiration, and in particular no spread of tumour cells along the needle track, this theoretical possibility must be considered.

Since our patients were usually treated very soon after aspiration with cytotoxic drugs, to which most of the tumours were at least initially sensitive, such a theoretical risk seemed to be of little importance compared with the advantages of rapid diagnosis which could be gained by the technique. In addition, it seems probable that a surgical procedure would be much more likely to cause tumour dissemination (Stewart, 1933; Dudgeon and Barrett, 1934; Martin and Stewart, 1936). However, this problem has been extensively studied by others and the risks when a narrow gauge needle is used appear to be negligible. Where needles of a gauge larger than 18 have been used there is a definite risk of spread, although the number of reports in the literature is small (Engzell, 1971). In 5–10 year follow-up studies of patients with cervical lymph node metastases, salivary pleomorphic adenoma or prostatic carcinoma diagnosed by fine needle aspiration, no evidence has emerged of local spread attributable to the biopsy (Engzell *et al.*, 1971*a*, *b*). Although many thousands of fine needle aspirates have been performed and reported in the literature, there are no definite reports of local tumour extension occurring as a result of the procedure (Eneroth *et al.*, 1967; Engzell *et al.* 1971*b*).

The risk of provoking more distant spread of tumour by aspiration has been examined in patients with mammary carcinoma (Robbins *et al.*, 1954; Berg and Robbins, 1962). In the group of patients whose tumours had been aspirated survival and tumour dissemination were very similar to another group of patients in whom standard biopsy procedures had been undertaken.

Thus the technique of fine needle aspiration appears to be safe, simple and reliable. There is a small incidence (4% in this series) of aspiration failure and occasionally problems of interpretation arise, although probably no more frequently than in the evaluation of histological sections. In all, there is much to recommend this technique and it deserves more widespread use than it currently has, although further experience with the cytological appearances of many tumours is required. It is particularly useful in a country such as Uganda where BL, in which cytological diagnosis is usually a simple matter readily learned by clinicians, accounts for more than half of all childhood malignancies and where problems of understaffed hospitals and a single pathology service for the whole country introduce significant delay into the establishment of a histological diagnosis.

Thanks are due to up-country physicians in Uganda for referring patients, to medical students who have assisted in the performance of aspirations and to Mr G. Buwule and Mrs P. Magrath who stained and mounted the smears. I am particularly grateful to Dr E. Shanbrom who first demonstrated the technique to me. Histological diagnoses were made by the pathologists of the Department of Pathology, Makerere University. Professors R. Owor and U. Engzell gave advice on the preparation of the manuscript and Mr A. Wamala and Mrs H. Sebambulidde provided secretarial assistance.

This work was supported by a grant from the Cancer Research Campaign (U.K.).

### REFERENCES

Ackerman, L. V. & del Regato, J. A. (1970) *Cancer, Diagnosis, Treatment and Prognosis*, 4th Ed. St Louis: C. V. Mosby.

Angstrom, T., Kjellgren, O. & Bergman, F. (1972) The Cytological Diagnosis of Ovarian Tumours by Means of Aspiration Biopsy. *Acta cytol.*, **26**, 336.

Aust, R., Stable, J. & Sterkrist, B. (1971) The Imprint Method for the Cytodiagnosis of Lymphadenopathies and of Tumours of the Head and Neck. *Acta cytol.*, **15**, 123.

Berard, C., O'Conor, G. T., Thomas, L. B. & Torloni, H. (1969) *Bull. Wld Hlth Org.*, **40**, 601.

Berg, J. W. & Robbins, G. F. (1962) A Late Look at the Safety of Aspiration Biopsy. *Cancer, N.Y.*, **15**, 826.

Berman, L. (1953) Malignant Lymphomas—their Classification and Relation to Leukemia. *Blood*, **8**, 195.

Bloch, M. (1967) A Comparative Study of Lymph Node Cytology by Puncture and Histopathology. *Acta cytol.*, **11**, 139.

BLUMING, A. Z. & TEMPLETON, A. C. (1971) Histological Diagnosis of Burkitt's Lymphoma: A Cautionary Tale. *Br. med. J.* i, 89.

BURIJAN, J., DJURIC, D. & TUFEGAZIC-LJALJERIC, J. (1960) Case of Hodgkin's Disease of Lungs Complicated by Bone Lesions and Diagnosed by Finding of Sternberg's Giant Cells in Sputum. *Srpski Arh. celok Lek.*, **88**, 99. Abstract in *Exerpta Med.*, (1971) Sect. 15, **14**, 480.

BURKITT, D. P. (1970) In *Burkitts Lymphoma.* Ed. D. P. Burkitt, and D. H. Wright. Edinburgh: E. & S. Livingstone, p. 6.

BUTLER, J. J. (1969) In *Comparative Morphology of Hemopoietic Neoplasms.* NCI Monogr. 32, Washington: U.S. Govt Printing Office. p. 237.

BUTLER, J. J. (1970) In *Leukemia-Lymphoma.* Chicago: Year Book, Med. Publishers Inc. p. 123.

CARDOZO, LOPES P. (1967) The Cytological Diagnosis of Lymph Node Punctures. *Am. J. clin. Path.*, **8**, 1964.

DAHLGREN, S. (1967) Aspiration Biopsy of Intrathoracic Tumours. *Acta path. mictobiol. scand.*, **70**, 566.

DAHLGREN, S. & LIND, B. (1969) Transthoracic Needle Biopsy or Bronchoscopic Biopsy. *Scand. J. resp. Dis.*, **50**, 265.

DAHLGREN, S. & LIND, B. (1972) Comparison between Diagnostic Results Obtained by Transthoracic Needle Biopsy and Sputum Cytology. *Acta cytol.*, **16**, 53.

DUDGEON, L. S. & BARRETT, N. R. (1934) The Examination of Fresh Tissues by the Wet-film Method. *Br. J. Surg.*, **22**, 4.

ENEROTH, C. M., FRANZEN, S. & ZAJICEK, J. (1967) Cytologic Diagnosis on Aspirates from 1000 Salivary Gland Tumours. *Acta otolaryng*, Suppl., **224**, 168.

ENGZELL, U. (1971) In *Aspiration Biopsy of Metastatic Carcinoma in Lymph Nodes—Clinical and Experimental Investigations.* Stockholm.

ENGZELL, U., JAKOBSON, P. A., SIZERDSON, A. & ZAJICEK, J. (1971*a*) Aspiration Biopsy of Metastatic Carcinoma of Lymph Nodes of the Neck. *Acta otolar.*, **72**, 138.

ENGZELL, U., ESPOSTI, P. L., RUBIO, C., SIGURDSON, A. & ZAJICEK, J. (1971*b*) Investigation on Tumour Spread in Connection with Aspiration Biopsy. *Acta radiol.*, **10**, 385.

FERGUSON, R. S. (1930) Prostatic Neoplasms. Their Diagnosis by Needle Puncture and Aspiration. *Am. J. Surg.*, **9**, 507.

FRANZEN, S., GIERTZ, G. & ZAJICEK, J. (1960) Cytological Diagnosis of Prostatic Tumours by Transrectal Aspiration Biopsy. A Preliminary Report. *Br. J. Urol.*, **32**, 193.

GODWIN, J. (1964) Cytological Diagnosis of Aspiration Biopsies of Solid or Cystic Tumours. *Acta cytol.*, **8**, 206.

GLEDHILL, E. Y., SPIGGS, J. B. & BINFORD, C. H. (1949) Needle Aspiration in the Diagnosis of Lung Carcinoma. Report of Experience with 75 Aspirations. *Am. J. clin. Path.*, **19**, 235.

GUTHRIE, C. G. (1921) Gland Puncture as a Diagnostic Method. *Bull John Hopkins Hosp.*, **32**, 266.

LUCAS, P. F. (1955) Lymph Node Smears in the Diagnosis of Lymphoadenopathy, Review. *Blood*, **10**, 1030.

MAGRATH, I. T., ZIEGLER, J. L. & TEMPLETON, A. S. (1973) A Comparison of Clinical and Histopathological Features of Childhood Lymphoma in Uganda. *Cancer, N.Y.*, In the press.

MARSAN, C. & BERTINI, B. (1960) La place du methodes cytologiques dans la diagnostic des tumeurs du sein. *Path. Biol.*, **8**, 343.

MARTIN, H. E. & ELLIS, E. B. (1934) Aspiration Biopsy. *Surgery, Gynec. Obstet.*, **59**, 578.

MARTIN, H. E. & STEWART, F. W. (1936) Advantages and Limitations of Aspiration Biopsy. *Am. J. Roentg.*, **35**, 245.

MOORE, R. D. & REAGAN, J. W. (1953) A Cellular Study of Lymph Node Imprints. *Cancer, N.Y.*, **6**, 600.

MORRISON, M., SAMWICK, A. A., RUBINSTEIN, J., STICH, M. & LOEWE, L. (1952) Lymph Node Aspiration. *Am. J. clin. Path.*, **22**, 255.

NASIELL, M. (1967) Diagnosis of Lung Cancer by Aspiration Biopsy and a Comparison between this Method and Exfoliative Cytology. *Acta cytol.*, **11**, 114.

ROBBINS, G. F., BROTHERS III, J. H., EBERHART, W. F. & QUAN, S. (1954) Is Aspiration Biopsy of Breast Cancer Dangerous to the Patient? *Cancer, N.Y.*, **7**, 774.

SHARP, G. S. (1931) The Diagnosis of Primary Carcinoma of the Lung by Aspiration. *Am. J. Cancer*, **15**, 863.

STEWART, F. (1933) The Diagnosis of Tumors by Aspiration. *Am. J. Path.*, **9**, 801.

STRUM, S. B., PARK, J. K. & RAPPAPORT, H. (1970) Observation of Cells Resembling Sternberg—Reed Cells in Conditions other than Hodgkin's Disease. *Cancer, N.Y.*, **26**, 176.

SYMMERS, W. ST C. (1968) Survey of the Eventual Diagnosis in 600 Cases Referred for a Second Histological Opinion after an Initial Biopsy Diagnosis of Hodgkin's Disease. *J. clin. Path.*, **21**, 650.

TRIBE, C. R. (1965) Cytological Diagnosis of Breast Tumours by the Imprint Method. *J. clin. Path.*, **18**, 31.

ULTMANN, J. E., KAPROWSKA, I. & ENGLE, R. L. (1954) A Cytological Study of Lymph Node Imprints. *Cancer, N.Y.*, **11**, 507.

VON HAAM, E. (1962) A Comparative Study of the Accuracy of Cancer Cell Detection by Cytological Methods. *Acta cytol.*, **6**, 508.

WRIGHT, D. H. (1967) In *The Treatment of Burkitt's Lymphoma.* U.I.C.C. Monograph, **8**, p. 14.

WRIGHT, D. H. (1970) In *Burkitt's Lymphoma.* Ed. D. P. Burkitt and D. H. Wright. Edinburgh: E. & S. Livingstone. p. 82.

ZAJICEK, J., FRANZEN, S., JAKOBSON, P., RUBIO, C. & VASGAARD, B. (1967) Aspiration Biopsy of Mammary Tumours in Diagnosis and Research. A Critical Review of 2,200 Cases. *Acta cytol.*, **11**, 169.